# An image database of *Drosophila melanogaster* wings for phenomic and biometric analysis

**DOI:** 10.1186/s13742-015-0065-6

**Published:** 2015-05-22

**Authors:** Anne Sonnenschein, David VanderZee, William R Pitchers, Sudarshan Chari, Ian Dworkin

**Affiliations:** 1 Genetics Graduate Program, Michigan State University, East Lansing, MI 48824 USA; 2 BEACON Center for the Study of Evolution in Action, Michigan State University, East Lansing, MI 48824 USA; 3 Department of Integrative Biology, Michigan State University, East Lansing, MI 48824 USA; 4 Department of Biology, McMaster University, Hamilton, Ontario, L8S 4K1 Canada

**Keywords:** Wing shape, *Drosophila*, Geometric morphometrics, Computer vision, Phenomics, Mutants

## Abstract

**Background:**

Extracting important descriptors and features from images of biological specimens is an ongoing challenge. Features are often defined using landmarks and semi-landmarks that are determined *a priori* based on criteria such as homology or some other measure of biological significance. An alternative, widely used strategy uses computational pattern recognition, in which features are acquired from the image *de novo*. Subsets of these features are then selected based on objective criteria. Computational pattern recognition has been extensively developed primarily for the classification of samples into groups, whereas landmark methods have been broadly applied to biological inference.

**Results:**

To compare these approaches and to provide a general community resource, we have constructed an image database of *Drosophila melanogaster* wings - individually identifiable and organized by sex, genotype and replicate imaging system - for the development and testing of measurement and classification tools for biological images. We have used this database to evaluate the relative performance of current classification strategies. Several supervised parametric and nonparametric machine learning algorithms were used on principal components extracted from geometric morphometric shape data (landmarks and semi-landmarks). For comparison, we also classified phenotypes based on *de novo* features extracted from wing images using several computer vision and pattern recognition methods as implemented in the Bioimage Classification and Annotation Tool (BioCAT).

**Conclusions:**

Because we were able to thoroughly evaluate these strategies using the publicly available *Drosophila* wing database, we believe that this resource will facilitate the development and testing of new tools for the measurement and classification of complex biological phenotypes.

**Electronic supplementary material:**

The online version of this article (doi:10.1186/s13742-015-0065-6) contains supplementary material, which is available to authorized users.

## Background

Understanding the causes and consequences of phenotypic variation is a unifying goal across many biological disciplines. One aim of phenomics is to comprehensively measure this variation. However, biological traits are complex and multidimensional and this presents challenges for both measurement and analysis [1]. The complete ‘phenome’ of an individual includes more phenotypes than can realistically be measured and the most informative subset of measurable features is not necessarily known, even for specific traits [2]. Manually selected features benefit from prior knowledge of the biological system, whereas computationally selected image properties are generally optimized for discrimination between groups. However, it is not clear how these strategies compare in their classification of images into groups (sex, genotype, species) or in their potential to derive broader biological inferences.

**Fig. 1:**
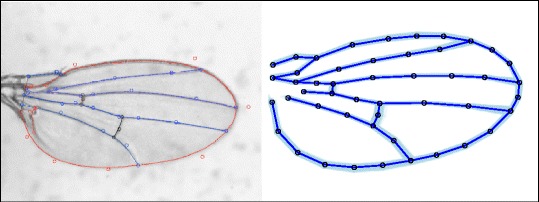
Wing landmarks and semi-landmarks. **a** Example wing image from *D. melanogaster* that has been splined using WINGMACHINE. **b** After landmark and semi-landmark data is extracted, data is translated (centered to origin), scaled by centroid size and superimposed (Procrustes superimposition for landmarks) data all lies in a common subspace. Image represents 50 individual configurations from specimens to demonstrate some of the variation among individuals

Geometric morphometrics and computational pattern recognition represent very different strategies for extracting and quantifying phenotypes from image data. Geometric morphometrics measures shape by using homologous landmarks (or curves) across specimens as features [3, 4]. Methodologically, these landmarks are determined *a priori* based on biological considerations of both homology and potential informativeness. Information about the shape of the specimen is extracted from the configuration by removing variation in size, location and orientation of the specimen, resulting in an explicit geometric representation of shape (Fig. 1) [5–7].

Computational pattern recognition represents a school of alternative approaches, in which features are extracted from image data with computer vision tools [8, 9]. Pattern recognition uses features such as the statistical distribution of pixels, or descriptions of texture or edges. A subset of informative features is generally selected based on an objective function, such as the classification of samples into groups, often using machine learning techniques [9]. The degree of informativeness of these features is usually assessed by cross-validation. Whereas geometric morphometrics requires a comprehensive understanding of the biological relevance and evolutionary history of the feature, computational pattern recognition can be applied without prior knowledge and can also detect informative patterns that are not visually perceptible [9].

Both geometric morphometrics and computational pattern recognition have practical applications in biological research. There have been varying levels of success using two-dimensional and three-dimensional cranial-facial morphometric phenotypes to infer the genetic causes of disease [10–12] and to track disease progression [13]. Morphometrics and computational pattern recognition have also been successfully used with machine learning algorithms to classify complex morphological phenotypes by species (e.g. [14–16]). Similarly, computer vision and pattern recognition have been crucial in the development of tools for the related field of biometrics [17], which uses phenotypes to distinguish individuals. Biometrics tools may be useful for interpreting phenomics data, thereby extending the amount of informative variation that can be extracted from biological images.

A potential biological application for biometrics is the interpretation of *Drosophila* wing shape. Wing shape is an established model system for phenomics [1, 18], the genetic basis of shape [19–21] and for phenotypic evolution [22, 23]. Although *Drosophila* wings can be evaluated qualitatively [24] or by metrics such as length and surface area [25], they are often measured within a geometric morphometric framework [14, 19, 26, 27]. Landmarks are based on vein intersections [26, 27] with semi-landmarks defining curves (Fig. 1) [14, 23]. Biometric facial recognition tools have had some success at classifying images of *Drosophila* wings into biological categories [28, 29]. The ‘eigenface’ method, which is a classic technique for facial recognition, has been modified into ‘eigenwings’ using features extracted from *Drosophila* wings to classify individuals by their sex [29]. Another facial recognition method that uses a genetic algorithm to select texture features [30] has also been used with similar goals [28–30], with up to a 94 % successful classification rate [29].

The success of facial recognition programs that rely on texture features instead of vein positioning raises the questions of what other features might also be useful for classifying *Drosophila* wings and how tools that are already used in biometrics may be applied to phenomics datasets. However, our ability to evaluate different approaches − whether for classification or biometric identification, as well as for long-term goals of further biological inference − remains limited by the lack of open databases of images for comparison.

In this article, we describe the creation and implementation of a database of wing images from *Drosophila melanogaster* for the development and testing of such methods. The database was designed to include multiple levels of replication encompassing both biological and technical variation. It allows the assessment of variation and classification by genotype, sex and individual identity (right and left wings from the same fly). To introduce sources of technical noise common to biological images, it includes several images of each wing, captured on various microscopes and at multiple magnifications.

Using landmark and semi-landmark measurements extracted from images in this database, we have analyzed the relative success of a number of machine learning algorithms at classifying *Drosophila* genotype and sex. We compare the success of these methods with the performance of the same classifier algorithms using features extracted by the Bioimage Classification and Annotation Tool (BioCAT), a pattern-recognition program designed for image analysis [31]. The database of images, landmark data and all source code have been made publicly available to serve as a resource for the testing and development of biometrics tools.

## Data description

**Fig. 2:**
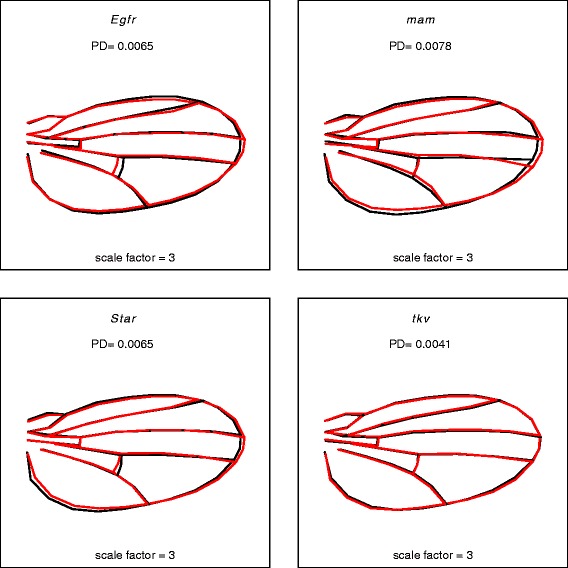
Magnitude and direction of the effect of each mutation (*red*) relative to Samarkand wild type (*black*). Magnitudes are in units of Procrustes distance (PD), which for this (tangent approximation) is equivalent to the Euclidean distance between the mean vector of each mutant and the Samarkand (SAM) wild type. The vectors of shape differences are magnified three-fold to enhance the clarity of the effects

**Fig. 3:**
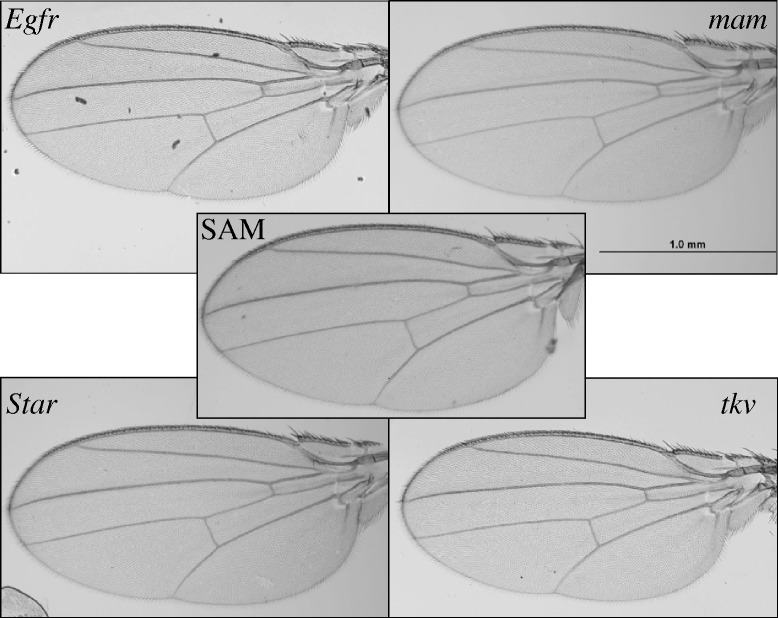
Representative images from the database. From right top corner counter-clockwise: *mastermind*, *Epidermal growth factor receptor*, *Star* and *thickveins. mastermind*, *Egfr* and *Star* mutations are all homozygous lethal and *thickveins* has a qualitative defect as a homozygote. As heterozygotes, they are qualitatively indistinguishable from the Samarkand (SAM) wild type (center)

The *Drosophila* wing database comprises a large number of high-quality wing images and contains both biological and technical variation. Sources of biological variation include genotype (there are four mutant genotypes (listed in Table 1) in the wild-type background of Samarkand (SAM), as well as the SAM wild-type background itself). In addition, sex and within-individual (left and right wings) variation is included. There are 100-130 individual samples for each combination of biological variables (Table 2). The mutant genotypes included in the database are heterozygous loss-of-function mutations for the genes that encode the Epidermal growth factor receptor (*Egfr*), mastermind (*mam*), Star (*S*) and thickveins (*tkv*) (see Table 1 for allele information, Fig. 2 for the relative impact of each mutation on phenotype and Methods for additional details). As heterozygotes, these mutations all have quantitative effects on shape, although they are qualitatively indistinguishable from the wild-type background (Fig. 3). The mutations represent perturbations of multiple signaling pathways: for example, *tkv* is a receptor kinase in the Transforming growth factor-β (TGF-β) pathway [32] and *mam* is a transcription factor in the Notch signaling pathway [33]. *Egfr* and *Star* genetically interact as *Star* modulates signaling through the Egfr pathway [32, 34]. These specific mutations were selected because previous studies have shown that when heterozygous, they have a range of quantitative effects on wing shape [27].

**Table 1:** *Drosophila* allele information

Bloomington stock number	Gene name	Gene symbol	Allele name
10385	*Epidermal growth factor receptor*	*Egfr*	P{lacW}*Egfr*^*k05115*^
14189	*mastermind*	*mam*	P{SUPor-P}*mam*^*kG02641*^
10418	*Star*	*S*	P{lacW}*S*^*k09530*^
14403	*thickveins*	*tkv*	P{SUPor-P}*tkv*^*KG01923*^

**Table 2:** *Drosophila* wings dissected by sex and genotype

	Female	Male
***Egfr***	116	118
***mam***	106	130
**Samarkand (SAM)**	107	100
***Star***	115	111
***tkv***	116	116

**Fig. 4:**
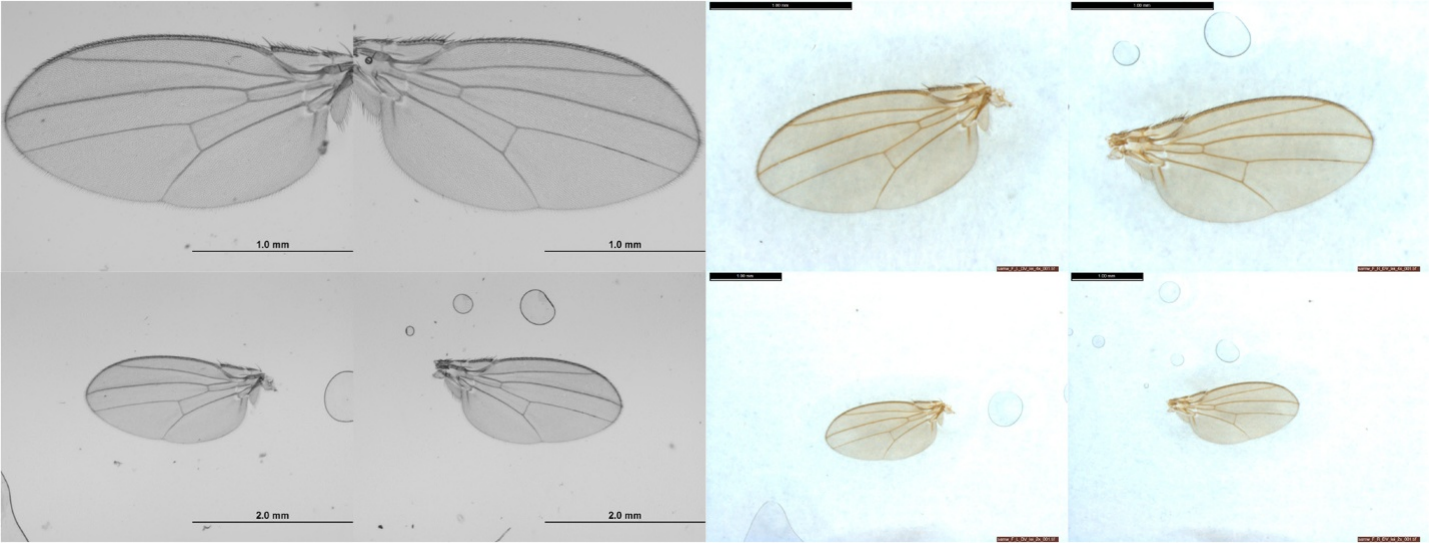
Left and right wings from the same female (SAM) fly, imaged four times. Top left are images taken on Olympus BX51 microscope at 40× magnification, top right are taken on Leica M125 at 40× magnification. Bottom left and right are images taken at 20× magnification

To allow researchers to compare various classification algorithms across a range of technical conditions, the measurements were subject to technical variation including the microscope and software used to capture images and the magnification setting of the microscope. Each wing in the database was imaged on two different microscope models, at both 40× and 20× magnification, so each wing in the database was imaged a total of four times (see Fig. 4: left and right wings from the same fly imaged under all four technical variation conditions). Also included in the database is landmark and semi-landmark coordinate information extracted from all images in the database using WINGMACHINE software [14], so information extracted from these images can be compared with existing standards for wing analysis. We also repeated the morphometrics analysis (landmarking, fitting curves (splining), editing splines and superimposition) for a small subset of the wing images (50 left and right female wings from two genotypes), to provide information on the technical variation in this process.

## Analyses

### Classification based on geometric morphometric data shows a high degree of accuracy across several supervised machine learning approaches

**Fig. 5:**
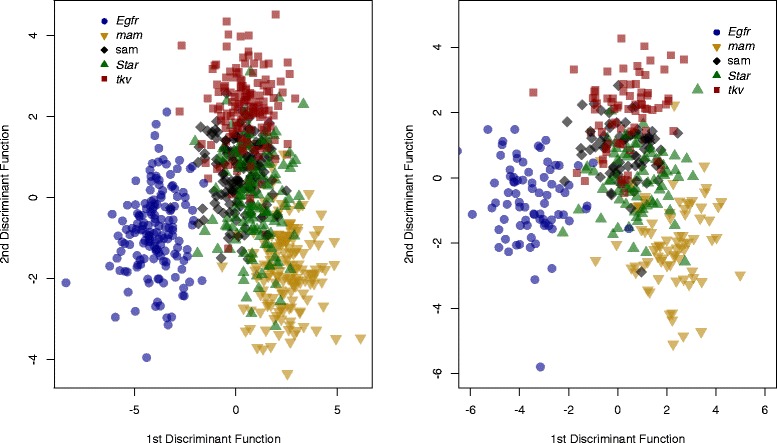
Separation of specimens using landmark data using linear discriminant analysis. Separation of specimens for each of the five genotype by linear discriminant analysis (LDA) in training set (left panel) and testing set (right panel), plotting the first discriminant function by the second discriminant function. This includes data for both males and females, but averaged (left and right wings) per specimen

Although the primary goal of this project was to develop the image database, we also wished to provide future users with some baseline data to evaluate classifiers. We used a wide range of supervised machine learning algorithms in R (version 3.1.0 [35]) for classification based on the landmark and semi-landmark data extracted from the images. All data for this analysis was from images taken on an Olympus BX51 microscope at 40× magnification. The data analyzed included the 58 principal components of shape generated from the landmark and semi-landmark coordinates (representing all non-zero eigenvalues). We chose algorithms to represent a wide range of models; standard errors were estimated by re-sampling training and testing sets. When classifying wings within a common genotype (SAM) by sex, all algorithms except for quadratic discriminant analysis (QDA) were able to predict the sex of a test set with more than 95 % accuracy (Table 3). When classifying wings by both genotype and sex, linear discriminant analysis (LDA), flexible discriminant analysis (FDA) and mixture discriminant analysis (MDA) were all able to correctly categorize test data with 85 % accuracy or higher. Support vector machines (SVM) and neural networks were also accurate with over 80 % of wings (Table 3). The high accuracy of most methods, especially of LDA (Fig. 5), suggests that classifications using this data, based on both sex and genotype, are robust to assumptions of linearity and common covariance matrices between factors (genotype and sex) [36].

**Table 3:** Classification accuracy of machine learning algorithms using landmark and semi-landmark data

Algorithm	Sex (± Standard error)	Genotype (± Standard error)
LDA	98.2 % (±1.6)	86.1 % (±1.5)
QDA	81.5 % (±6.4)	68.7 % (±2.2)
FDA	98.2 % (±1.6)	86.0 % (±1.5)
MDA	98.1 % (±1.6)	84.8 % (±1.6)
Bagging	93.3 % (±2.9)	57.6 % (±2.9)
Random forest	94.6 % (±2.7) 100 trees	74.9 % (±2.1) 1,000 trees
SVM	96.8 % (±2.1) sigmoid	83.8 % (±1.6) radial
Neural network (size 10)	98.3 % (±1.6)	81.2 % (±2.2)
KNN	98.3 % (±1.5) k = 4	59.3 % (±2.1) k = 32

### Computational feature detection and sub-setting for classification using BioCAT

We tested several methods of classification using the image analysis software BioCAT [31], which allows combinations of feature selectors, extractors and classifiers. Using the Fisher feature selection criterion, we tested several combinations of features and classification algorithms (Additional file 1: Supplementary Methods). After training a random forest classifier with 50 FeatureJ Hessians [37] extracted from a training set of wing images, we were able to classify individuals by sex (in a common genotype) in a test set of wing images with 85 % accuracy (Table 4). Classification of wing images by genotype (within sex) had an accuracy of only up to 52 %, although this is higher than the 20 % success rate that would be expected for random classification.

**Table 4:** Classification accuracy of machine learning algorithms compared with BioCAT

Classification	Algorithm	Hessian	Shape	Shape + Size
Sex	Random forest (10)	85.0 %	92.3 % (±3.7)	94.7 % (±2.6)
	Random forest (1,000)	85.0 %	96.1 % (±2.2)	95.9 % (±2.1)
	SVM	81.7 %	99.0 % (±1.2)	99.0 % (±1.2)
Genotype	Random forest (10)	52.0 %	43.3 % (±3.5)	44.7 % (±3.7)
	Random forest (1,000)	46.7 %	69.1 % (±3.4)	70.2 % (±2.8)
	SVM	43.3 %	75.1 % (±2.8)	75.8 % (±2.7)

Hessian column represents accuracy of classifications based on Hessian features extracted with BioCAT. Shape column represents classification accuracy based on landmarks and semi-landmarks, not including centroid. Shape + size represents classification accuracy based on landmarks and semi-landmarks, including centroid

### Comparisons between BioCAT and geometric morphometric descriptors for classification

BioCAT feature selectors act on raw images and therefore had access to both shape and size information for wings, whereas morphometric analyses were performed after scaling by centroid. When the parameter for centroid size was included with landmark and semi-landmark coordinates in morphometric analysis, the relative effectiveness of different algorithms was largely the same (Table 4), although classification accuracy generally increased for both sex and genotype.

**Fig. 6:**
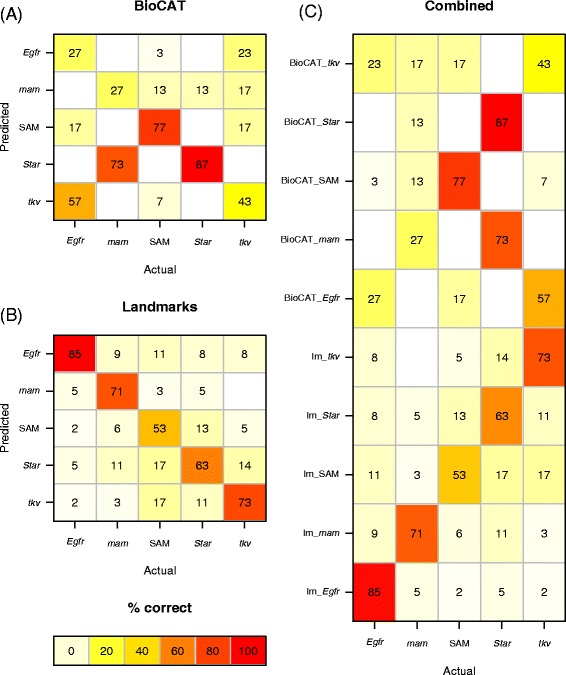
Confusion matrices. Heatmap of confusion matrices from classification (random forest) using features extracted using BioCAT **(a)** compared with landmark and semi-landmark data **(b)**. The data in **(a)** and **(b)** is shown together in **(c)** to facilitate comparison. Numbers represent percentage of correct classifications. lm_* represent the landmark/semi-landmark data. BioCAT features were mis-classified more consistently as some genotypes, e.g. mis-classification of *mam* mutants as *Star* **(a)**. This pattern is not evident in the classification using the landmark data **(b)**. Scale represents frequency of classification

Classification based on features extracted by BioCAT and those using landmarks and semi-landmark coordinates differed in the distribution of classification errors between genotypes. BioCAT classified some genotypes far more consistently than others and errors frequently skewed towards a particular genotype (Fig. 6). Notably, *mastermind* was misclassified as *Star* 90 % of the time (27/30 mis-identifications in the test set). There is no similar trend of *Star* and *mam* phenotype mis-identification evident in classifications based on landmarks and semi-landmarks (Fig. 6).

### Comparisons between BioCAT and geometric morphometric descriptors for classification across datasets

Both geometric morphometric methods and BioCAT were able to classify images by sex across sources of technical variation (i.e. images taken on different microscopes). Geometric morphometric methods showed very little loss in accuracy when classifying wings at the same magnification across microscopes (where an LDA was trained on images taken on the Olympus at 40× magnification and tested on images from the Leica microscope at the same magnification). However, accuracy dropped substantially from 98.2 % to 81.2 % when the LDA was trained on images taken on the Olympus at 40× magnification and tested on images taken on the same microscope at 20× magnification (Table 5). Images from each microscope and magnification were superimposed separately and simultaneous superimposition might substantially increase the accuracy of classification across datasets.

**Table 5:** Classification accuracy of machine learning algorithms compared with BioCAT for predicting sex

Method	Training images	Testing images	Sex (± Standard error)
BioCAT	Olympus 40×	Olympus 40×	85.0 %
	Olympus 40×	Olympus 20×	50.0 %
	Olympus 40× cropped	Olympus 20× cropped	50.0 %
	Leica 40× cropped	Leica 40× cropped	93.0 %
	Olympus 40× cropped	Leica 40× cropped	73.7 %
	Olympus & Leica 40× cropped	Olympus 40× cropped	73.3 %
	Olympus & Leica 40× cropped	Leica 40× cropped	86.0 %
Landmarks	Olympus 40× landmarks	Olympus 40× landmarks	98.2 % (±1.6)
	Olympus 40× landmarks	Olympus 20× landmarks	81.2 % (±1.4)
	Leica 40× landmarks	Leica 40× landmarks	97.8 % (±0.69)
	Olympus 40× landmarks	Leica 40× landmarks	79.1 % (±1.3)

Machine learning algorithms using landmark and semi-landmark features, compared with Hessian features extracted by BioCAT, trained and tested across microscopes and magnifications

BioCAT was not able to make accurate classifications across datasets using unedited images. When trained on images taken on the Olympus at 40×, it uniformly classified wings in images taken on the Olympus at 20× magnification as males (using Hessian features and a random forest classifier). Using images cropped to the same dimensions as used for measuring splines (cropping images was also necessary for geometric morphometric analysis), BioCAT still had difficulty classifying across magnifications, but was able to correctly identify sex from wing images taken at the same magnification on the Leica microscope with 73.7 % accuracy (Table 5). Interestingly, BioCAT performed better on images taken on the Leica - when both trained and tested on images taken on the Leica at a common magnification, it classified images by sex with 93 % accuracy, relative to 85 % accuracy when classifying images from the Olympus microscope (Table 5).

## Discussion

Although this database is primarily intended to serve as a resource for the development and testing of measurement tools, we also investigated whether the image collection could provide insights into the comparative effectiveness of existing pattern recognition and morphometrics methods. In particular, we compared *a priori* biologically informed landmark data as features analyzed within a geometric morphometrics framework with *de novo* feature extraction, identification and optimization. Using both types of features, we evaluated the classification of wings by genotype and by sex and the accuracy of various statistical learning methods. The performance of the classifiers based on landmark data was generally superior with respect to classifying test data. For a number of reasons, this success must be considered within the context of the methods examined in this study. The availability of the database now provides a test bed for further refinement.

Computational pattern recognition and morphometrics software are likely to extract different features. In addition to considering how well geometric morphometric approaches compare to ‘computer vision’ *de novo* feature extraction (see below), it is also worth comparing the efficiency with which the feature data can be obtained. The WINGMACHINE pipeline was designed with a single goal and has been optimized for extracting landmark and semi-landmark data from *Drosophila* wings. By contrast, BioCAT was designed (and therefore chosen for this study) for its accessibility and flexibility, which allow it to be immediately applied to raw wing images. The FeatureJ features extracted with BioCAT describe image texture [31], whereas a geometric morphometric approach uses biologically defined, homologous landmarks and curves as features, defined by vein intersections and outlines [14]. Extracting the large number of landmarks and semi-landmarks used in this study is laborious for most biological systems. Even using the WINGMACHINE pipeline requires multiple stages of image processing, some manual landmark acquisition and manual correction of splines after automated fitting. By contrast, both feature extraction and classification using BioCAT were performed without *a priori* annotation or editing. Thus, despite the overall success in classification using the landmark and semi-landmark data, the efficiency of acquiring the data must also be considered for other studies.

Perhaps unsurprising given the different nature of the features extracted, the machine learning algorithms that were most able to classify wings (for sex and genotype) using BioCAT’s Hessian features differed from those that were most able to classify wings using landmark and semi-landmark data. Whereas SVMs consistently performed better than random forests for classification using morphometric data, the reverse was true using features extracted with BioCAT (Table 4). Classifications based on landmarks and semi-landmarks were also substantially improved by increasing the number of trees in the random forest from 10 (the BioCAT default) to 1,000 trees. BioCAT classification success was unaffected or slightly lowered by an increase in the number of trees.

The Drosophila wing database contains large numbers of wing images representing multiple genotypes. It also includes several built-in controls for technical variation that should make it amenable to the development of biometric classification tools. Using the landmark and semi-landmark data extracted with WINGMACHINE, wings can be classified by sex and genotype with high levels of accuracy. We were also able to classify wings by sex and genotype with relatively good accuracy using texture features extracted by the computer vision software BioCAT. We hope that this database will serve as a resource for research into the sources of variation contributing to wing shape and for the development and testing of measurement tools for image-based phenomics.

## Methods

### Fly genetics and sample preparation

Fly stocks were obtained from the Bloomington Stock Center. These lines include wing mutations in the genes *Epidermal growth factor receptor* (*Egfr*), *mastermind* (*mam*), *thickveins* (*tkv*) and *Star* (*S*; see Table 1 for allele and stock information). All four mutations are caused by insertions of P-element transposable elements, each marked with a mini-*w*^*+*^ resulting in partial rescue of wild-type red eyes. The wild-type strain used as a background was an isogenic Samarkand (SAM), marked with a *w*^*−*^ mutation to enable identification of the mutant alleles [38].

Each P-element-bearing strain was initially introgressed into SAM by repeatedly backcrossing into the SAM background genotype (as described in [27]). These have since been maintained heterozygous balanced over a CyO (also in the SAM background) with the exception of the *tkv* mutant, which was maintained as a homozygote. Before initiating the experiment, these flies were maintained for one generation in an incubator (Percival Model : I41VLC8 set to 24 °C, 65 % relative humidity and a 12-hour light/dark cycle) to acclimatize them to the environment. Under these same growth conditions, the lines carrying mutant alleles were then backcrossed for two additional generations into the SAM wild-type background prior to rearing flies for data collection for the database. Because of the extensive back-crossing, each mutant-bearing strain is close to co-isogenic to the SAM wild type, with the exception of the focal allele and a small genomic fragment in linkage disequilibrium to that allele.

For each mutant strain, populations were expanded in five replicate bottles. Each bottle contained 10 mutant males (red-eyed) crossed to 20 SAM virgin females (white-eyed). This experimental design was also applied to the SAM control, with 10 SAM males and 20 SAM virgin females. The flies were allowed to lay eggs for 4 days, after which the adults were discarded. After 7 days, paper towel was added to the bottles to soak up excess moisture and provide additional substrate for pupation. From days 14-18, emerging flies were phenotyped (based on the *w*^*+*^ marker) and sexed. They were stored separately by sex and genotype in microtubes containing 70 % ethanol at room temperature for wing dissections. Fly wings were dissected in phosphate buffered saline (PBS) and mounted on slides in a solution of 70 % glycerol and 30 % PBS. All wings dissections were performed by the same person (AS). If one wing was torn or damaged, both wings from that fly were discarded.

### Imaging

Each wing was imaged at 20× and 40× magnification on both an Olympus BX51 and Leica M125 microscope, using the DP controller (V.3,1,1208) and Leica App Suite (V.3) imaging software respectively. Two individuals imaged wings, one using the Olympus microscope (AS) and the other the Leica (DV) microscope. All images have unique names, using the format ‘genotype_sex_side_microscope_magnification_fly-number’. If images contained tears or folds in the wing or indicated errors in dissection or mounting, all images from that fly were discarded.

### Geometric morphometric data acquisition and preparation

Images were first converted to grayscale and cropped with the Gnu Image Manipulation Program (GIMP, version 2.8 [39]) in batches using the David’s Batch Processor plugin (version 1.1.8 [40]). Two starting landmarks were manually labeled at the humeral break and alula notch, using tpsDig (version 2.17 [41]). For more details on re-sizing and cropping, see Additional file 1: Supplementary Methods. WINGMACHINE (Wings version 3.7.2 [14]) was used to generate wing splines, which were manually reviewed and adjusted as necessary. CPR (version 1.01r [42]) was used to scale wings by centroid size, perform a Procrustes superimposition and extract landmark and semi-landmark coordinates. Further details on processing of this data are available in Additional file 1: Supplementary Methods. All further statistical analysis was done in R (version 3.1.0 [35]) on images of wings taken at 40× magnification on the Olympus BX51 microscope. Scripts can be found at the Dworkin Lab github page [43] and together with the data at GigaDB [44].

Procrustes coordinate values and centroid for left and right wings from the same fly were averaged using the R *plyr* package (V.1.8.1). In total, this included 12 two-dimensional landmarks and 36 semi-landmarks. However, because of image registration, scaling and Procrustes superimposition, four dimensions do not contain any information. Furthermore, the semi-landmarks are constrained to slide along a curve and therefore have approximately one degree of freedom. This results in approximately 58 dimensions of potential data. Thus, the first 58 principal components contributing to shape (excluding centroid) were extracted and used for all further analyses.

### Morphometric analysis

Two-thirds of the samples from each genotype were defined as the training set and one-third as a testing set. These were used to train and test ‘lda’ and ‘qda’ functions from the MASS package (V. 7.3-33), ‘mda’ and ‘fda’ functions from the *mda* package (V. 0.4-4), the ‘bagging’ function from the *adabag* package (V.3.2), random forest from the *randomForest* package (using 500 trees, version 4.6-7), the ‘svm’ function from the *e1071* package (version 1.6-3) and a neural network from the *nnet* package (V. 7.3-8). K-nearest neighbors (KNN) from the *class* package (V. 7.3-10) was also tested, using k values from 1 to 100. Confidence intervals were approximated by re-sampling the training and testing sets over 1,000 repetitions. All functions were used with default arguments, with the exception of ‘svm’, ‘knn’ and random forest. ‘svm’ was optimized for kernel function shape and ‘knn’ for the value of k. Random forests were tested over a range of 10-1,000 trees.

SVM and random forest (using 10 trees and 1,000 trees) were repeated on several subsets of the original dataset, using only left wings (prior to averaging) and left-female wings, to facilitate comparisons with the BioCAT results. The same analysis was also used to classify wings by sex, using only wings from the SAM (wild-type) genotype.

### BioCAT analysis

For the BioCAT analysis [14], we used a Fisher feature selector to identify 50 eigenvalues of the Hessian matrix [37] from 144 left male and female wings (77 each) from the SAM genotype. BioCAT applied these features to train an SVM classifier and two random forest classifiers: one with 10 trees and one with 1,000 trees. These models were used to annotate 30 male and 30 female left SAM wings that were not included in the training set. Annotation accuracy was determined by counting the number of correct and incorrect classifications. Although BioCAT allows for cross-validation during combined training/testing, the quantity and size of our data made re-sampling for confidence intervals infeasible. This process was repeated for classification by genotype using 70 left-female wings from each of the five genotypes as a training set for classification by genotype. The genotype classification models were tested on 30 left-female wings from each genotype. Images used as training and testing sets have been organized with their respective models at the database [44].

### Availability and requirements

- Project name: Source code from an image database of *Drosophila melanogaster* wings for phenomic and biometric analysis.
- Project home page: https://github.com/gigascience/paper-sonnenschein2015.
- Operating systems: Windows, OS X, Linux.
- Programming language: R.
- Other requirements: None.
- License: GPLv3.
- Any restrictions to use by non-academics: None.

## Availability of supporting data

Additional information on image processing for WINGMACHINE, parameters used for machine learning in R and BioCAT analysis is available in Additional file 1: Supplementary Methods. The *Drosophila* wing database is publicly available at the *GigaScience* GigaDB repository [44].

## Additional file

Additional file 1:
**Supplementary Methods.**

## Abbreviations

BioCAT: BIOimage classification and annotation tool
Egfr: Epidermal growth factor receptor
FDA: Flexible discriminant analysis
KNN: K-nearest neighbors
LDA: Linear discriminant analysis
mam: Mastermind
MDA: Multiple discriminant analysis
QDA: Quadratic discriminant analysis
S: Star
SAM: Samarkand
SVM: Support vector machine
tkv: Thickveins
