# Supplementary material for: An image database of Drosophila melanogaster wings for phenomic and biometric analysis
Source: Gigascience. 2015 May 22;4:25. doi: 10.1186/s13742-015-0065-6 (PMC4942975; doi:10.1186/s13742-015-0065-6)
Supplement: Additional file 1: — Supplementary Methods. [file 13742_2015_9065_MOESM1_ESM.doc]

**Additional file 1: Supplementary Methods**

1. Image processing for WINGMACHINE and CPR

2. Supplementary methods for BioCAT

**1. Image processing for WINGMACHINE and CPR**

**1.1 Cropping wing images**

Wing images taken at 2x on the Olympus BX51 microscope and at both 2x and 4x on the Leica M125 microscope were cropped (using the David's Batch Processor plugin in GIMP [1]) for analysis with WINGMACHINE. All images taken on the Olympus were initially 1360 x 1024 pixels (px). 2x images were cropped to 750 x 555px. All images on the Leica were initially 1392 x 1040px. Leica 4x images were cropped to 960 x 718px, except for a small subset in which the orientation of the wing required larger dimensions and these were cropped to 1200 x 898px. Leica 2x images were primarily cropped to 500 x 374px, except for a small subset that were cropped to 600 x 449px. The dimensions that were used for each image are indicated in the files 'Leica_2X_coords.tsv' and 'Leica_2X_coords.tsv' in the *GigaScience* GigaDB repository [2].

**1.2 Landmarking wing images with tpsUtil and tpsDig**

Wing images were resized for landmarking to the dimensions 632 x 480 pixels. A.tps file was created using tpsUtil (version 1.58 [3]), and landmarked in tpsDig2 [3]. The.tps file was converted to an.asc file using a custom python script.

**1.3 Splining wing images**

Images for splining were resized to the dimensions 316 x 240px. All WINGMACHINE splines were manually checked and edited.

**1.4 Superimposition in CPR**

WINGMACHINE output was superimposed in CPR. After superimposition and sliding of semi-landmarks, the most proximal semi-landmark on vein 4 was removed as described in [4]. Data for the first five principal components were visually checked as a scatterplot and outliers caused by incorrect spline alignment were manually corrected.

**1.5 Identifying scale in cropped images**

Scale mm/px values were manually calibrated in ImageJ version 1.48 [5].

**2. Supplementary methods for BioCAT**

This section includes a subset of informative results from testing various BioCAT settings with different subsets of wings. These subsets of the wing images, and the classification models, are available at the *GigasScience* GigaDB repository [2]. For these trials, training was done using BioCAT's 'Training only' option, followed by annotation using the 'test set'. In all cases, only left wings were used from the Olympus 40x dataset. Unless otherwise indicated, default settings were used. Data that was entered into the confusion matrices for Figure 6 are indicated (see code in [2]).

**2.1 Classifying by sex within Samarkand genotype, using approximately equal numbers of each class for training and testing.** Overall, the best results were with Hessian features combined with Random forest classifiers. F, female; M, male; RF, random forest; SAM, Samarkand; SVM, support vector machine.

| **Features** | **Classifier** | **Training set** | **Testing set** | **Success rate** | **Notes** |
| --- | --- | --- | --- | --- | --- |
| Structure  30 | RF  10 trees | 68 M SAM wings  71 F SAM wings  (Sex training set 1) | 15 M SAM wings  15 F SAM wings  (Sex test set 1) | 14/15 F correct  11/15 M correct |  |
| Structure  50 | RF  10 trees | 68 M SAM wings  71 F SAM wings  (Sex training set 1) | 15 M SAM wings  15 F SAM wings  (Sex test set 1) | 14/15 F correct  11/15 M correct |  |
| Laplacian  30 | RF  10 trees | 68 M SAM wings  71 F SAM wings  (Sex training set 1) | 15 M SAM wings  15 F SAM wings  (Sex test set 1) | 12/15 F correct  8/15 M correct |  |
| Laplacian  30 | SVM  linear | 68 M SAM wings  71 F SAM wings  (Sex training set 1) | 15 M SAM wings  15 F SAM wings  (Sex test set 1) | Very low |  |
| Stats  30 | RF  10 trees | 68 M SAM wings  71 F SAM wings  (Sex training set 1) | 15 M SAM wings  15 F SAM wings  (Sex test set 1) | 13/15 F correct  12/15 M correct |  |
| Derivatives  30 | SVM  linear | 68 M SAM wings  71 F SAM wings  (Sex training set 1) | 15 M SAM wings  15 F SAM wings  (Sex test set 1) | 11/15 F correct  14/15 M correct |  |
| Hessian 30 | SVM  linear | 68 M SAM wings  71 F SAM wings  (Sex training set 1) | 15 M SAM wings  15 F SAM wings  (Sex test set 1  and Sex test set 2) | Test 1:  13/15 F correct  14/15 M correct  Test 2:  8/15 F correct  15/15 M correct |  |
| Structure 50 | RF  10 trees | 68 M SAM wings  71 F SAM wings  (Sex training set 1) | 15 M SAM wings  15 F SAM wings  (Sex test set 1  and Sex test set 2) | Test 1:  12/15 F correct  11/15 M correct  Test 2:  10/15 F correct  15/15 M correct |  |
| Structure 50 | SVM  linear | 68 M SAM wings  71 F SAM wings  (Sex training set 1) | 15 M SAM wings  15 F SAM wings  (Sex test set 1  and Sex test set 2) | Test 1:  11/15 F correct  13/15 M correct  Test 2:  8/15 F correct  15/15 M correct |  |
| Hessian 50 | RF  10 trees | 68 M SAM wings  71 F SAM wings  (Sex training set 1) | 15 M SAM wings  15 F SAM wings  (Sex test set 1  and Sex test set 2) | Test 1:  13/15 F correct  13/15 M correct  Test 2:  10/15 F correct  15/15 M correct | Best  85%  Table 4  Table 5 |
| Hessian 50 | SVM  linear | 68 M SAM wings  71 F SAM wings  (Sex training set 1) | 15 M SAM wings  15 F SAM wings  (Sex test set 1  and Sex test set 2) | Test 1:  11/15 F correct  14/15 M correct  Test 2:  9/15 F correct  15/15 M correct | 81.70%  Table 4 |
| Hessian 50 | RF  1,000 trees | 68 M SAM wings  71 F SAM wings  (Sex training set 1) | 15 M SAM wings  15 F SAM wings  (Sex test set 1  and Sex test set 2) | Test 1:  13/15 F correct  13/15 M correct  Test 2:  10/15 F correct  15/15 M correct | 85%  Table 4 |

**2.2 Classifying by genotype - Samarkand vs. mutant phenotypes (Egfr, mam, S, tkv) with varying numbers of trees.** The best results were again achieved with Hessian combined with Random forest. SVM linear performed much better than SVM with alternate kernel shapes. RF, random forest; SAM, Samarkand; SVM, support vector machine.

| **Features** | **Classifier** | **Training set** | **Testing set** | **Success rate** | **Notes** |
| --- | --- | --- | --- | --- | --- |
| Hessian 30 | RF 10 trees | Genotype training 1  327 mutant females  77 SAM females | Genotype test 1  15 of each mutant  15 SAM | Identifies all wings as  Mutant |  |
| Hessian 30 | SVM linear | Genotype training 1  327 mutant females  77 SAM females | Genotype test 1  15 of each mutant  15 SAM | 9/15 SAM wrong  6/60 mutants wrong |  |
| Hessian 30 | SVM radial | Genotype training 1  327 mutant females  77 SAM females | Genotype test 1  15 of each mutant  15 SAM | Identifies all wings as  Mutant |  |
| Hessian 30 | SVM sigmoid | Genotype training 1  327 mutant females  77 SAM females | Genotype test 1  15 of each mutant  15 SAM | Very low accuracy |  |
| Structure 30 | RF 10 trees | Genotype training 1  327 mutant females  77 SAM females | Genotype test 1  15 of each mutant  15 SAM | Very low accuracy |  |
| Structure 30 | SVM linear | Genotype training 1  327 mutant females  77 SAM females | Genotype test 1  15 of each mutant  15 SAM | 2 mutants wrong  9/15 SAM wrong |  |
| Structure 30 | SVM radial | Genotype training 1  327 mutant females  77 SAM females | Genotype test 1  15 of each mutant  15 SAM | Very low accuracy |  |
| Hessian 50 | SVM linear | Genotype training 2  77 mutant females  77 SAM females | Genotype test 2  15 mutants total (3-4 of each)  15 SAM | Test 1:  6/15 SAM correct  13/15 mutants correct  Test 2:  2/15 SAM correct  15/15 mutants correct | Equal  representation in train/test sets  greatly  improved  accuracy |
| Structure 50 | SVM linear | Genotype training 2  77 mutant females  77 SAM females | Genotype test 2  15 mutants total 15 SAM | Test 1:  12/15 SAM correct  7/15 mutant correct  Test 2:  13/15 SAM correct  10/15 mutant correct |  |
| Structure 50 | RF 10 trees | Genotype training 2  77 mutant females  77 SAM females | Genotype test 2  15 mutants total 15 SAM | Test 1:  10/15 SAM correct  11/15 mutants correct  Test 2:  9/15 SAM correct  12/15 mutants correct |  |
| Hessian 50 | RF 10 trees | Genotype training 2  77 mutant females  77 SAM females | Genotype test 2  15 mutants total 15 SAM | Test1:  11/15 SAM correct  13/15 mutants correct  Test 2:  11/15 SAM correct  12/15 mutants correct |  |

**2.3 Classifying by genotype - all genotypes (females).** Egfr, epidermal growth factor receptor; mam, mastermind; RF, random forest; SAM, Samarkand; SVM, support vector machine; tkv, thickveins.

| **Features** | **Classifier** | **Training set** | **Testing set** | **Success rate** | **Notes** |
| --- | --- | --- | --- | --- | --- |
| Hessian 50 | RF 10 trees | Genotype training 3  70 wings of each  genotype, females | Genotype test 3  15 wings of each  genotype, females  x2 test sets | EgfrT1: 20%; 3 correct; 2 SAM; 10 tkv  EgfrT2: 33.3%; 5 correct; 3 SAM; 7 tkv  MamT1: 20%; 3 correct; 12 star  MamT2: 33.3%; 5 correct; 10 star  SAMT1: 86.7%; 1 mam; 1 Egfr; 13 correct  SAMT2: 66.7%; 3 mam; 10 correct; 2 tkv  StarT1: 100%; 15 correct  Star T2: 73.3%; 11 correct; 4 mam  TkvT1: 26.7%; 4 correct; 4 mam; 5 Egfr; 2 SAM  TkvT2: 60%; 9 correct; 2 Egfr; 1 mam; 3 SAM | 52%  combined  Table 4  Figure 6 |
| Hessian 50 | SVM linear | Genotype training 3  70 wings of each  genotype, females | Genotype test 3  15 wings of each  genotype, females | EgfrT1: 1 correct; 1 SAM; 13 tkv  EgfrT2: 1 correct; 2 mam; 1 SAM; 11 tkv  MamT1: 1 correct; 14 Star  MamT2: 2 correct; 13 Star  SAMT1: 11 correct; 1 Egfr; 3 mam  SAMT2: 4 correct; 6 Egfr; 4 mam; 1 tkv  StarT1: 14 correct; 1 mam  StarT2: 13 correct; 1 mam; 1 tkv  TkvT1: 6 correct; 3 Egfr; 6 SAM  TkvT2: 13 correct; 2 mam | 44%  overall  Table 4 |
| Structure 50 | SVM linear | Genotype training 3  70 wings of each  genotype, females | Genotype test 3  15 wings of each  genotype, females | EgfrT1: 3 correct; 2 SAM; 10 tkv  EgfrT2: 1 correct; 1 mam; 5 SAM; 8 tkv  MamT1: 15 correct  MamT2: 1 correct; 14 Star  SAMT1: 11 correct; 1 Egfr; 3 mam  SAMT2: 9 correct; 5 Egfr; 1 mam  StarT1: 14 correct; 1 mam  StarT2: 14 correct; 1 mam  TkvT1: 6 correct; 2 Egfr; 7 SAM  TkvT2: 9 correct; 1 Egfr; 5 SAM | 45.3%  overall |
| Structure 50 | RF 10 trees | Genotype training 3  70 wings of each  genotype, females | Genotype test 3  15 wings of each  genotype, females | EgfrT1: 5 correct; 3 SAM; 7 tkv  EgfrT2: 3 correct; 1 mam; 3 SAM; 8 tkv  MamT1: 3 correct; 12 Star  MamT2: 3 correct; 12 Star  SAMT1: 13 correct; 1 Egfr; 1 mam  SAMT2: 10 correct; 3 Egfr; 2 tkv  StarT1: 14 correct; 1 mam  StarT2: 13 correct; 2 mam  TkvT1: 1 correct; 6 Egfr; 4 mam; 1 SAM  TkvT2: 6 correct; 5 Egfr; 4 SAM | 47.3 %  overall |
| Hessian 50 | RF 1,000 trees | Genotype training 3  70 wings of each  genotype, females | Genotype test 3  15 wings of each  genotype, females | EgfrT1: 0 correct; 2 SAM; 13 tkv  EgfrT2: 2 correct; 4 SAM; 9 tkv  MamT1: 1 correct; 14 Star  MamT2: 2 correct; 13 Star  SAMT1: 12 correct; 1 Egfr; 1 mam  SAMT2: 10 correct; 1 Egfr; 2 mam; 2 tkv  StarT1: 15 correct  StarT2: 13 correct; 2 mam  TkvT1: 6 correct; 2 Egfr; 5 mam; 2 SAM  TkvT2: 9 correct; 1 Egfr; 1 mam; 4 SAM | 47% overall  Table 4 |

**2.4 Classifying by sex across microscopes/magnifications.** Egfr, epidermal growth factor receptor; F, female; M, male; mam, mastermind; RF, random forest; SAM, Samarkand; SVM, support vector machine; tkv, thickveins.

| **Features** | **Classifier** | **Training set** | **Testing set** | **Success rate** | **Notes** |
| --- | --- | --- | --- | --- | --- |
| Hessian 50 | RF 10 trees | 68 M SAM wings  71 F SAM wings  Olympus, 40x mag  uncropped | Test 1  15 M SAM wings  15 F SAM wings  Test 2  15 M SAM wings  15 F SAM wings  Olympus 20x  uncropped | Identifies 100% as male  50% overall | Table 5 |
| Hessian 50 | RF 10 trees | 68 M SAM wings  71 F SAM wings  Olympus, 20x mag  uncropped | Test 1  15 M SAM wings  15 F SAM wings  Test 2  15 M SAM wings  15 F SAM wings  Olympus 20X  uncropped | Test 1:  13/15 F correct  10/15 M correct  Test 2:  12/15 F correct  15/15 M correct  83.3% overall |  |
| Hessian 50 | RF 10 trees | 68 M SAM wings  71 F SAM wings  Olympus, 40x mag cropped for splining | Test 1  15M SAM wings  15 F SAM wings  Test 2  15M SAM wings  15 F SAM wings  Olympus 40x cropped for splining | Test 1  12/15 F correct  13/15 M correct  Test 2  10/15 F correct  15/15 M correct  83.3% overall |  |
| Hessian 50 | RF 10 trees | 68 M SAM wings  71 F SAM wings  Olympus, 40x mag cropped for splining | Test 1  15M SAM wings  15 F SAM wings  Test 2  15M SAM wings  15 F SAM wings  Olympus 20x cropped for splining | Test 1  0/15 F correct  14/15 M correct  Test 2  1/15 F correct  15/15 M correct  50% overall | Table 5 |
| Hessian 50 | RF 10 trees | 68 M SAM wings  71 F SAM wings  Olympus, 40x mag cropped for splining | Test 1  15 M SAM wings  15 F SAM wings  Test 2  15M SAM wings  12 F SAM wings  Leica 40x  cropped for splining | Test 1  7/15 F correct  14/15 M correct  Test 2  7/12 F correct  14/15 M correct  73.7% overall | Table 5 |
| Hessian 50 | RF 10 trees | 68 M SAM wings  71 F SAM wings  Leica, 40x mag cropped for splining | Test 1  15 M SAM wings  15 F SAM wings  Test 2  15 M SAM wings  12 F SAM wings  Leica 40x  cropped for splining | Test 1  15/15 F correct  15/15 M correct  Test 2  12/12 F correct  11/15 M correct  93.0% overall | Table 5 |
| Hessian 50 | RF 10 trees | 68 M SAM wings  71 F SAM wings  Leica, 40x mag cropped for splining | Test 1  15M SAM wings  15 F SAM wings  Test 2  15M SAM wings  15 F SAM wings  Olympus 40x cropped for splining | Test 1  14/15 F correct  0/15 M correct  Test 2  14/15 F correct  0/15 M correct |  |
| Hessian 50 | RF 10 trees | Mixed microscopes  15 males Oly 4x  15 males Lei 4x  15 females Oly 4x  15 females Lei 4x | Test 1  15M SAM wings  15 F SAM wings  Test 2  15M SAM wings  15 F SAM wings  Olympus 40x | Test 1  13/15 F correct  8/15 M correct  Test 2  15/15 F correct  8/15 M correct | Table 5  73.3% |
| Hessian 50 | RF 10 trees | Mixed microscopes  15 males Oly 4x  15 males Lei 4x  15 females Oly 4x  15 females Lei 4x | Test 1  15M SAM wings  15 F SAM wings  Test 2  15M SAM wings  15 F SAM wings  Leica 40x | Test 1  13/15 F correct  14/15 M correct  Test 2  9/12 F correct  13/15 M correct | Table 5  86.0% |

**2.5 Classifying by genotype using Leica 4x images.** Egfr, epidermal growth factor; mam, mastermind; RF, Random forest; SAM, Samarkand; tkv thickveins.

| **Features** | **Classifier** | **Training set** | **Testing set** | **Success rate** | **Notes** |
| --- | --- | --- | --- | --- | --- |
| Hessian 50 | RF 10 trees | Genotype train Leica  64 wings from each genotype  Leica 4x  Cropped prior to landmark, splining  960 x 718 pixels | Genotype test Leica  All from Leica 4x  Test set 1  15 wings from each genotype  Test set 2  15 wings from each genotype | EgfrT1:  9/15 correct  6/15 SAM  EgfrT2:  12/15 correct  3/15 SAM  MamT1:  1/15 correct  10/15 Star  4/15 tkv  MamT2:  2/15 correct  2/15 Egfr  3/15 SAM  4/15 Star  4/15 tkv  SAMwT1:  14/15 correct  1/15 mam  SAMwT2:  8/15 correct  7/15 mam  StarT1:  14/15 correct  1/15 mam  StarT2:  15/15 correct  TkvT1:  2/15 correct  4/15 mam  2/15 SAMw  7/15 Star  TkvT2:  3/15 correct  3/15 mam  9/15 Star | 53.3%  accurate overall.  This isn't substantially higher than the accuracy using the Olympus images (52% overall), unlike sex predictions. |

**References**

1. David's Batch Processor version 1.1.8. 2015. http://members.ozemail.com.au/~hodsond/dbp.html. Accessed 24 April 2015.
2. Sonnenschein A, VanderZee D, Pitchers WR, Chari S, Dworkin I. An image database of *Drosophila melanogaster* wings for phenomic and biometric analysis.
3. tpsUtil. 2015. http://life.bio.sunysb.edu/morph/soft-utility.html. Accessed 24 April 2015.
4. Pitchers W, Pool JE, Dworkin I. Altitudinal clinical variation in wing size and shape in African Drosophila melanogaster: one cline or many? Evolution. 2013;67:38-42
5. Image J. 2015. http://imagej.nih.gov. Accessed 24 April 2015.
